# Thirteenth International Medical Congress Transactions

**Published:** 1901-11

**Authors:** A. Chanthard

**Affiliations:** General Secretary. Paris


*Editor Buffalo Medical Journal:*

### THIRTEENTH INTERNATIONAL MEDICAL CONGRESS TRANSACTIONS.

*Editor Buffalo Medical Journal:*

Sir—The general secretary has the honor to inform the members of the Thirteenth International Congress of Medicine that the printing and forwarding of the general volume and of the seventeen records of the sections is entirely finished now.

Any member who, by mistake, should not have received the volumes where he is entitled to, is hereby requested to address his reclamation to the editors of the Congress, Mrt. Masson & Co., 120 Boulevard Saint Germain, Paris.

After December 31, 1901, any reclamation will not be acknowledged. A. CHANTHARD, General Secretary.

Paris, September 9, 1901.

